# 970. Assessing the burden of *Acinetobacter baumannii* and *Candida auris* among ventilated patients in Maryland: A statewide period prevalence survey of two urgent threats

**DOI:** 10.1093/ofid/ofad500.2464

**Published:** 2023-11-27

**Authors:** Anthony Harris, Lisa Pineles, J Kristie Johnson, Lyndsay M O’Hara, Lauren Leigh Smith, Indira French, Jamie Rubin, Rebecca Perlmutter, Ashley Heller, Liore Klein, David Blythe, Elisabeth Vaeth

**Affiliations:** University of Maryland School of Medicine, Baltimore, MD; University of Maryland School of Medicine, Baltimore, MD; University of Maryland School of Medicine, Baltimore, MD; University of Maryland School of Medicine, Baltimore, MD; University of Maryland, Baltimore, MD; University of Maryland School of Medicine, Baltimore, MD; MDH, Baltimore, Maryland; Maryland Department of Health, Baltimore, Maryland; Maryland Department of Health, Baltimore, Maryland; Maryland Department of Health, Baltimore, Maryland; Maryland Department of Health, Baltimore, Maryland; Maryland Department of Health, Baltimore, Maryland, Baltimore, Maryland

## Abstract

**Background:**

To date, only one statewide prevalence survey has been performed for *Acinetobacter baumannii* (2009) in the United States, and no statewide prevalence survey has been performed for *Candida auris*. We aimed to determine the prevalence of *A*. *baumannii* and *C. auris* among mechanically ventilated patients in Maryland.

**Methods:**

The Maryland MDRO Prevention Collaborative performed a statewide point prevalence survey of mechanically ventilated patients admitted to acute care hospitals (ACH) and long-term care (LTC) facilities between March 7, 2023, and June 8, 2023. Surveillance cultures were obtained from all mechanically ventilated patients. Sputum, peri-anal, and arm/leg cultures were cultured for *A. baumannii* and antibiotic susceptibility testing was performed. Axilla/groin cultures were tested by PCR for *C. auris*.

**Results:**

One hundred percent of all eligible healthcare facilities participated. 482 ventilated patients were included in the analysis. *A. baumannii* was cultured from at least one body site in 148/482 (31%) patients, CRAB was identified in 88/482 (18%) patients and *C. auris* in 31/470 (7%) patients. LTC patients were more likely to be colonized with *A. baumannii* (RR 7.7, 95% CI 5.1-11.5, p< .0001), with CRAB (RR 5.5, 95% CI 3.4-8.9, p< .0001) and *C. auris* (RR 2.0, CI 0.99-3.9, p=.05) compared to acute care hospital patients (Figure 1). Nine (29%) *C. auris-*positive patients were previously unknown to the Maryland Department of Health. Susceptibilities for *A. baumannii* are in Table 1.

**Figure 1:**
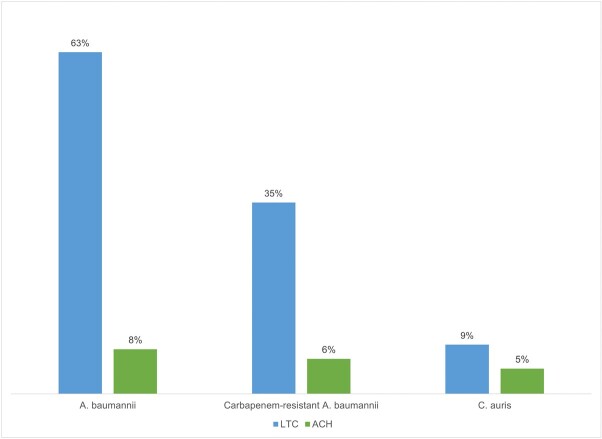
Prevalence of A. baumannii, carbapenem-resistant A. baumannii, and C. auris stratified by type of facility.

**Figure 2.**
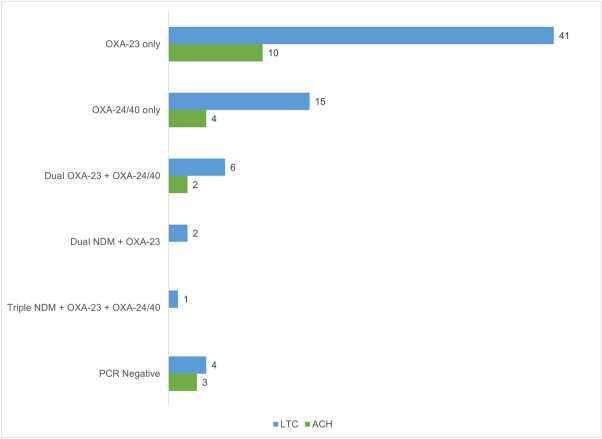
Resistance Mechanisms in Carbapenem-Resistant Isolates (N=88)

**Table 1:**
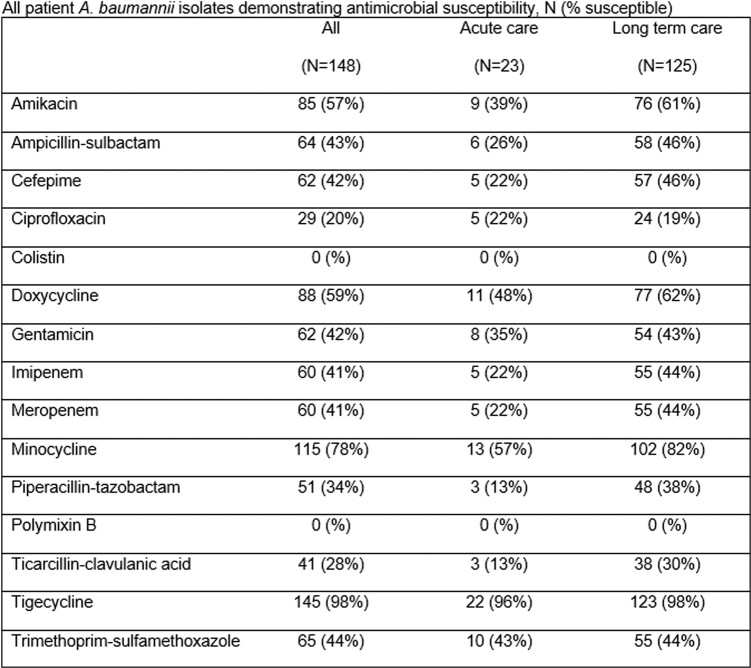
Antimicrobial Data: All patient A. baumannii isolates demonstrating antimicrobial susceptibility, N (% susceptible)

**Conclusion:**

*A. baumannii*, CRAB and *C. auris* colonization are common among mechanically ventilated patients in both ACH and LTC facilities. Both pathogens were significantly more common in long-term care facilities than acute care facilities. Ventilated long-term care patients are an extremely high-risk population for emerging pathogens and surveillance and infection prevention efforts should be targeted to these facilities.

**Disclosures:**

**Anthony Harris, MD, MPH**, Merck: Grant/Research Support|UpToDate: Infection Control Editor

